# Pediatric Acute Megakaryoblastic Leukemia with a GATA2 Mutation and Monosomy 7: A Case Report and Clinical Management Challenges

**DOI:** 10.3390/reports8030153

**Published:** 2025-08-21

**Authors:** Gowri Joshi, Astil Jisho Anto, Md Maaz Mallick, Gwan Yong Lim, Łukasz Hutnik

**Affiliations:** 1 Pediatric Hematology ED Student Scientific Club, Department of Pediatric Hematology and Oncology, Medical University of Warsaw, 02-091 Warsaw, Poland; 2 Department of Pediatric Hematology and Oncology, Medical University of Warsaw, 02-091 Warsaw, Poland

**Keywords:** case report, acute megakaryoblastic leukemia, chemoresistance, pediatric leukemia, myelodysplastic syndrome, GATA2 mutation, monosomy 7, hematopoietic stem cell transplantation

## Abstract

**Background and Clinical Significance**: Acute megakaryoblastic leukemia (AMKL) is a rare and aggressive hematologic malignancy. The presence of genetic abnormalities often increases the complexity of AMKL. Among these, patients with monosomy 7 constitute a high-risk group associated with a poorer prognosis and greater chemoresistance. We report the case of a 10-year-old boy who had AMKL along with monosomy 7 and familial GATA2 deficiency. The case highlights the diagnostic and therapeutic challenges faced, as well as the critical importance of early genetic screening and timely hematopoietic stem cell transplantation (HSCT). **Case Presentation**: A 10-year-old boy presented with easy bruising and pancytopenia. AMKL was diagnosed with the help of a bone marrow biopsy and immunophenotyping. Genetic testing showed a GATA2 mutation and monosomy 7. Two induction cycles with daunorubicin and cytarabine were administered but failed to eliminate residual disease. The patient also developed pneumonia of a fungal origin. HSCT was delayed due to liver toxicity and elevated minimal residual disease (MRD). Azacitidine and venetoclax stabilized the disease, thereby allowing for successful haploidentical HSCT. The patient achieved complete remission with full donor chimerism. **Conclusions**: This case emphasizes the importance of early molecular diagnostics in pediatric AMKL. Identifying GATA2 mutations and monosomy 7 early can help guide risk stratification and the timing of HSCT. Multimodal therapy, which includes the use of infection control and targeted agents, is important for improving the outcomes in high-risk patients.

## 1. Introduction and Clinical Significance

Acute megakaryoblastic leukemia (AMKL) is a rare and aggressive malignancy that arises from the myeloid hematopoietic lineage, characterized by the malignant proliferation of megakaryoblasts. AMKL is most commonly associated with somatic GATA1 mutations, which contribute to 20–30% of AMKL cases. Rare cases are associated with germline GATA2 mutations and cytogenetic abnormalities such as monosomy 7 [1,2]. While monosomy 7 frequently co-occurs in patients with GATA2 deficiency, it is rare for them to present with pediatric AMKL. GATA2 is a transcription factor that regulates the expression of genes critical for embryonic development, self-renewal, maintenance, and functionality of hematopoietic, lymphatic, and multipotent stem cells [1]. These rare genetic features are clinically significant, as they are linked to a significantly poorer prognosis, increased susceptibility to opportunistic infections, and reduced tolerance to standard chemotherapeutic regimens [3,4]. In pediatric patients with GATA2 deficiency and monosomy 7, early allogeneic hematopoietic stem cell transplantation (HSCT) is considered the only curative option because of the high risk of progression and a poor response to chemotherapy [4]. Additionally, granulomatous lymphocytic interstitial lung disease (GLILD), which is most often described in patients with common variable immunodeficiency but has also been reported in association with GATA2 deficiency [5], and its occurrence in pediatric AMKL is rare, adding to the diagnostic and therapeutic complexity.

We present the case of a 10-year-old boy diagnosed with AMKL, carrying a germline GATA2 mutation and a secondary clonal monosomy 7 in their bone marrow, which necessitated individualized chemotherapy, aggressive infection management, close clinical monitoring, and an early referral for hematopoietic stem cell transplantation (HSCT). The patient also developed GLILD, which was initially suspected to be a fungal infection. Given the atypical presentation and underlying genetic abnormalities, this case also emphasizes the importance of early genetic screening in pediatric patients with resistant or unusual forms of acute myeloid leukemia.

## 2. Case Presentation

**Figure 1:**
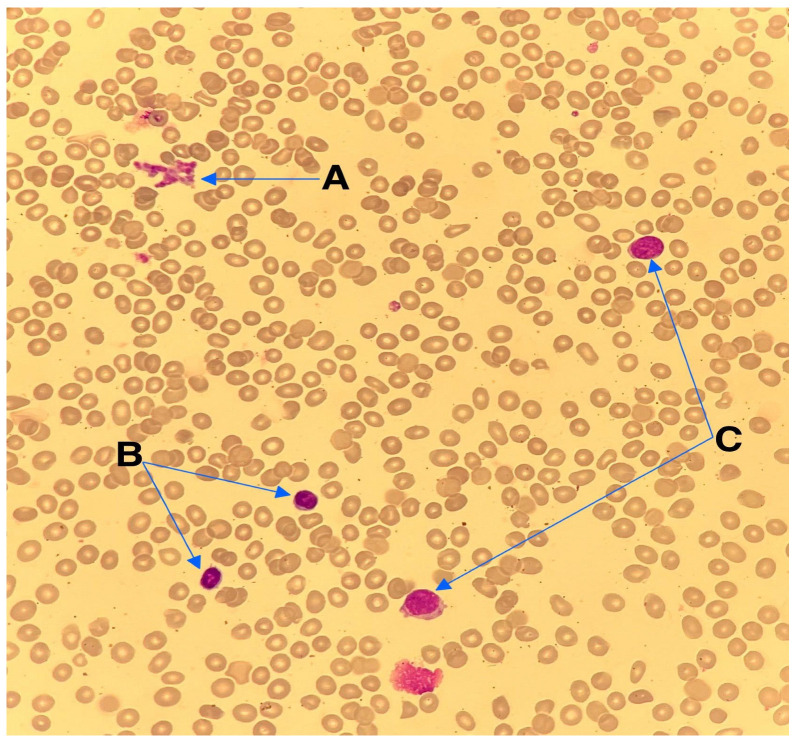
Wright–Giemsa staining of the bone marrow aspirate at a 40× magnification. (A) Platelet aggregation. (B) Lymphocytes. (C) Blast cells with a high nuclear-to-cytoplasmic ratio and cytoplasmic protrusions or “blobs” which resemble platelet budding, which suggests megakaryoblastic differentiation, a characteristic feature of AMKL.

A 10-year-old boy presented to the hospital with easy bruising, exercise intolerance, and a fever. He had had a prior cytomegalovirus infection and was admitted due to persistent abnormal blood counts. A physical exam showed pallor, petechiae, and bruises on his lower extremities and right elbow. Initial blood morphology tests showed a hemoglobin level of 5.1 g/dL (10–15.5 g/dL), 4.2 × 10^9^/L leukocytes (4.5–13.5 × 10^9^/L), 82 × 10^9^/L platelets (150–450 × 10^9^/L), and a neutrophil count of 0.64 × 10^9^/L (1.4–6.1 × 10^9^/L), indicating pancytopenia. Bone marrow aspiration was inconclusive due to severe artifacts. Although insufficient for a definitive morphological diagnosis, the smear showed occasional blast cells with cytoplasmic protrusions suggestive of megakaryoblastic differentiation (Figure 1); therefore, a trephine biopsy was performed. It showed hypocellular marrow (30–40%) with fibrosis, a reduced myeloid lineage, dysplastic megakaryocytes, and 10% weak CD34+ cells, suggesting myelodysplastic syndrome. Following the trephine biopsy, a histopathological and immunophenotypic analysis of the bone marrow was performed due to a suspicion of MDS, which confirmed AMKL, with CD61 positivity on numerous dysplastic megakaryocytes and CD71 positivity on the erythroid lineage. Genetic analysis revealed a germline GATA2 mutation and an acquired monosomy 7 in leukemic cells, confirmed by conventional karyotyping and FISH, placing the patient at a high risk. The patient’s family history was significant for maternal GATA2 deficiency; currently in her thirties, the patient’s mother remains asymptomatic. A germline status was inferred from the presence of an identical mutation in the patient’s asymptomatic mother. The mother was not considered a suitable haploidentical donor.

The patient underwent two induction cycles with a fixed-dose liposomal formulation of daunorubicin and cytarabine (123 IU/m^2^). One month after the first cycle, bone marrow evaluation revealed 2% blast cells and an MRD of 0.22%, as assessed by multiparameter flow cytometry according to institutional protocols, indicating incomplete remission. On day 1 of the second induction, the MRD was 0.62%, suggesting possible chemoresistance. Following the treatment, the patient developed prolonged pancytopenia and persistent fever. A chest CT showed signs of a pulmonary fungal infection, for which he was treated with amphotericin B and voriconazole.

**Figure 2:**
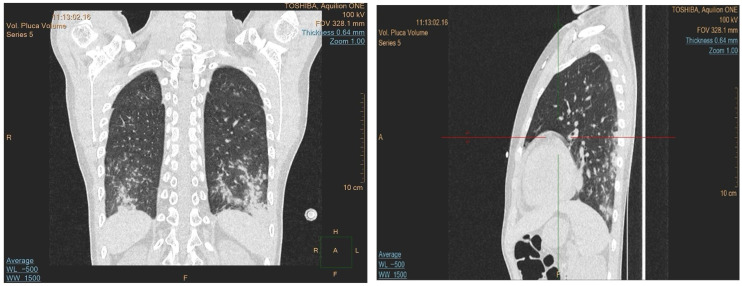
Chest CT scans with coronal and sagittal views confirming the presence of bilateral, diffuse ground-glass opacities mainly in the lower lobes, with reticular interstitial thickening.

Due to the progression of the lung lesions observed in the follow-up imaging, amphotericin B was switched to isavuconazole. The patient also received broad-spectrum antibiotics and transfusions. A sinus MRI was performed to search for other potential sites of fungal involvement, which revealed suspected fungal changes in the left frontal sinus; drainage was performed, but no fungal growth was detected in the cultures. As the fever persisted despite antifungal and antibiotic treatment, an immunological cause was suspected. A control CT revealed progression of the lung lesions despite the antifungal treatment, which raised the suspicion of granulomatous lymphocytic interstitial lung disease (GLILD) (Figure 2). Bronchoalveolar lavage was performed, and the recovered fluid was sent for histopathological analysis and when stained using a Grocott stain showed no signs of fungal involvement, and no acid-fast bacteria were observed. However, cytology of the specimen showed 56% lymphocytes, 29% macrophages, 14% neutrophils, and 1% eosinophils. These histopathological findings, along with ground-glass infiltrate in a chest CT, helped diagnose the patient with GLILD, and the patient was subsequently treated with rituximab.

Bone marrow evaluation showed an MRD of 0.06%, and the decision to proceed with HSCT was made while the patient was in hematological remission to avoid further complications.

The patient was transferred to the Hematopoietic Cell Transplantation unit of the University Children’s Hospital in Kraków to proceed with HSCT from a haploidentical donor (father). The donor was screened for the familial mutation and was found to be negative. In the control bone marrow biopsy performed before the transplant, the MRD was found to have increased to 0.93%. Taking into account the increased MRD, genetic mutations, and toxicity of the previous treatment, it was decided to postpone the transplant, and the patient was started on a cycle of azacitidine with venetoclax. Following treatment, the patient was transferred for haploidentical HSCT, which was preceded by a myeloablative BuFluTT + post-Cy conditioning regimen. The haploidentical HSCT from his father proceeded without complications. Post-transplant, he developed mucositis, cryptococcal meningitis, for which he was treated with amphotericin B (5 mg/kg dose) and fluconazole, CMV reactivation, and a Clostridioides difficile infection, all managed successfully. The engraftment criteria were met with WBC > 1000/μL by day +19 and ANC > 500/μL by day +20, following G-CSF administration. The patient’s platelet counts remained above 20,000/μL from day +55, when the final transfusion was performed as part of the planned procedures. The patient was discharged on day +73 post-transplant in a good general condition.

## 3. Discussion

GATA2 deficiency is a rare genetic disorder caused by germline haploinsufficiency of a transcription factor essential for vascular development, hematopoietic stem cell maintenance, and immunity development. GATA2 deficiency is autosomal dominant in nature and is associated with several abnormalities, such as cytopenias, recurrent infections, immunodeficiency, and myelodysplastic syndrome (MDS), with a high risk of transformation to acute myeloid leukemia (AML) [1].

The disruption of megakaryopoiesis caused by the GATA2 mutation increases the risk of developing AMKL [6]. The combination of GATA2 mutations and monosomy 7 catalyzes the leukemic transformation, which often leads to aggressive disease courses.

GATA2 mutation testing is not performed routinely in all patients with AML. It is considered in patients with MDS/AML who have monosomy 7 or other high-risk cytogenetic abnormalities such as unexplained or persistent cytopenias, recurrent or atypical infections, and a family history of bone marrow failure. GATA2 deficiency is also associated with extra-hematologic manifestations, including sensorineural hearing loss and pulmonary alveolar proteinosis [1,5]. Identifying a GATA2 mutation in these settings can help provide prognostic information, optimize the timing of HSCT, and warrant genetic counseling for the families affected.

Monosomy 7 is a relatively common cytogenetic abnormality found in patients who have GATA2 deficiency. Studies have shown that approximately 70% of children with GATA2 mutations present with monosomy 7. This chromosomal loss is associated with a higher risk of progression from MDS to AML. Additionally, GATA2 germline mutations are recognized as the leading hereditary contributor to MDS in adolescents with monosomy 7.

The presence of both these abnormalities often correlates with an early disease onset and higher risk of progression to AML and distinct molecular signatures that differentiate these patients from those with monosomy 7 alone. A study which focused on pediatric MDS revealed that GATA2 mutations are highly prevalent in those with monosomy 7, but the presence of the mutation itself does not affect the overall survival. The prognosis is primarily dictated by the presence of monosomy 7 [2].

Somatic GATA2 mutations are uncommon in AML, occurring in <5% of AML cases [7]. GATA2 mutations, especially ones affecting zinc finger domains, impair DNA binding and affect transcriptional activity [8]. This haploinsufficiency disrupts normal blood cell development and predisposes individuals to MDS/AML [9]. Research also indicates that GATA2 can act as a tumor suppressor, and its inactivation due to mutations can accelerate disease progression.

The patient was administered induction chemotherapy which included cytarabine and daunorubicin, which represents the most commonly used first-line regimen for AML, which is often referred to as the “7 + 3” protocol. However, the patient’s MRD levels did not significantly decrease after two inductions, indicating chemoresistance due to their genetic mutations. Therefore, the therapeutic strategy was adjusted accordingly.

In this case, chemoresistance likely arose from the coexistence of the GATA2 deficiency and monosomy 7, both of which are independently associated with a poor treatment response in myeloid malignancies. GATA2 plays a crucial role in hematopoietic stem cell generation and maintenance. While GATA2 deletion may increase the risk of developing MDS/AML, some studies show that the mutation results in a more robust induction of p53 after exposure to chemotherapeutic drugs. This would imply that GATA2 deletion increases p53-mediated apoptosis, which may help in decreasing chemoresistance [10]. However this case is more complex due to the additional cytogenetic abnormality of monosomy 7.

Monosomy 7 is one of the most frequent cytogenetic abnormalities and is found in both pediatric and adult myeloid malignancies. This chromosomal deletion causes the haploinsufficiency of several key tumor suppressors located on chromosome 7, such as EZH2, PMS2, HUS1, and NAMPT. The consequent loss of function contributes to impaired DNA repair, genomic instability, and impaired epigenetic regulation, causing resistance to standard chemotherapy [3,11].

This co-occurrence of the GATA2 mutation and monosomy 7 enhances the negative effect of the damage caused by each individually on the body, resulting in an accelerated transformation to a hematological malignancy and an increase in chemoresistance. Patients with this combination typically exhibit incomplete remission following standard treatment, as seen in our case, making HSCT the best curative option. The prevalence of these mutations strongly influenced the decision to shift from chemotherapy to HSCT, in alignment with the current guidelines.

The MRD is an important marker for monitoring the treatment response, predicting the relapse risk, and managing further treatment. A positive MRD indicates that leukemia cells are still present in the body after treatment. The MRD status prior to allo-HSCT is a strong prognostic factor for the overall survival, disease-free survival, and cumulative incidence of relapse [12]. Patients who were MRD-negative before transplantation showed better overall survival and a reduced relapse risk compared to MRD-positive patients.

Due to immunosuppression caused by the underlying disease and the treatment, the initial lung infiltrates observed on the CT were treated empirically with antifungals. However, the progression observed in follow-up imaging raised suspicion of GLILD, and the antifungals were switched to rituximab. GLILD is diagnosed based on a combination of compatible high-resolution CT findings, characteristic lymphoid and granulomatous histopathology, and exclusion of infection and malignancy [13]. While GLILD is primarily associated with common variable immunodeficiency, rare cases have linked it to GATA2 deficiency [5], making it an uncommon but clinically important pulmonary complication in this setting.

A critical reason we are presenting this case is to illustrate the role of HSCT. Early HSCT is recommended in patients with GATA2 mutations and monosomy 7 due to the high likelihood of disease progression and poor long-term outcomes with chemotherapy alone. The only curative treatment is allogeneic hematopoietic stem cell transplantation (HSCT) [5]. In this case, HSCT using a haploidentical donor was initially delayed due to two major concerns: an increase in the MRD to 0.93%, which could have resulted in a higher relapse risk, and the onset of grade 4 liver toxicity, likely a result of chemotherapy and the administration of rituximab used for the management of GLILD. As a result, the decision was made to delay HSCT until the patient’s condition improved and the leukemia was better controlled. This was achieved with a combination of azacitidine and venetoclax used as a bridging therapy before the HSCT [14].

In this case, venetoclax, which is a BCL-2 inhibitor, and azacitidine played a key role in reducing the MRD and stabilizing the disease before the transplant. Due to the high MRD and liver toxicity caused by the previous treatment, proceeding directly to HSCT carried a higher chance of relapse or adverse complications related to the transplant. This drug regimen is low-intensity while still being effective; it is increasingly being used in high-risk or chemoresistant AML, particularly in patients who have adverse cytogenetics or some treatment-related toxicities, where high-intensity chemotherapy is potentially harmful or contraindicated. The combination induces apoptosis in leukemic cells while minimizing the additional toxicity, making it a viable option as a bridging therapy before transplantation [14,15]. New studies have shown that the use of this combination improved pre-transplant disease control and also reduced the MRD, results that correlated with better transplant outcomes [16]. In this case, the successful reduction in the MRD and full donor chimerism after the transplant demonstrates the potential of this approach for use in complex and similar AML scenarios.

A study which analyzed pediatric patients with a GATA2 deficiency-related MDS reported a 5-year overall survival rate of 75% and disease-free survival rate of 70% post-HSCT. The prognosis was poorer in the presence of monosomy 7, with a disease-free survival rate of 67% compared to 90% in patients with a cytogenetically normal karyotype. Age also played a role in the prognosis, with younger patients having a better prognosis [4].

The patient received conditioning treatment according to the busulfan, fludarabine, thiotepa, and post-transplant cyclophosphamide (BuFluTT + post-Cy) protocol. This regimen is used in high-risk HSCT to optimize the efficacy, minimize the toxicity, and prevent graft-versus-host disease (GvHD). BuFluTT is myeloablative and takes advantage of the use of alkylating busulfan along with a combination of fludarabine and thiotepa to achieve disease control with the minimum toxicity. Research indicates that busulfan-based conditioning regimens, such as BuFlu, are associated with reduced transplant-related toxicity compared to traditional busulfan–cyclophosphamide (BuCy) regimens, without affecting the overall survival [17]. PTCy is an effective strategy for GvHD prophylaxis in haploidentical HSCT. It selectively targets alloreactive T cells post-transplantation, reducing the incidence of GvHD without negatively affecting the graft-versus-leukemia effect. This approach has favorable outcomes in pediatric patients undergoing haploidentical HSCT [18].

The post-transplant period was complicated due to the reactivations of CMV and BKV; the patient also developed a Clostridioides difficile infection and a cryptococcal infection. However, the patient was treated promptly, which eventually led to a negative MRD, and full donor chimerism was achieved by day +30. This underlines the critical role of early management of infectious and inflammatory complications post-HSCT.

In the most recent follow-up, the patient remained in a good general condition with no complications. The absence of post-transplant complications is a good sign; however the long-term prognosis remains uncertain due to the presence of GATA2 deficiency, which warrants regular check-ups and can cause extramedullary manifestations and immune dysfunction. The risk of a relapse cannot be excluded as 24–41% pediatric patients with AML may experience a disease relapse despite initial remission [19].

## 4. Conclusions

The presence of GATA2 deficiency and monosomy 7 significantly influences the prognosis and clinical management, necessitating early consideration of HSCT. This case highlights the importance of early genetic screening in pediatric patients with high-risk or chemoresistant hematological malignancies.

In this case, MRD monitoring provided critical insights for modification of treatment and the timing of HSCT. The case also shows how delays in HSCT can be prevented by using bridging therapies such as azacitidine and venetoclax, which help to stabilize the patient before transplantation and may improve the outcomes in similar cases. Timely diagnosis, tailored chemotherapy, and close monitoring for infections and inflammatory complications were important to achieve remission and stabilize the patient’s condition for HSCT, which is the treatment of choice in high-risk AMKL cases. GATA2 deficiency is also associated with extra-hematologic manifestations, including sensorineural hearing loss, pulmonary alveolar proteinosis, and increased susceptibility to atypical mycobacterial infections, all of which warrant long-term surveillance [1,5]. This case also highlights the importance of flexible treatment planning in the management of high-risk pediatric AML.

## Data Availability

The data used in this study may be obtained from the authors upon reasonable request due to privacy concerns.

## Abbreviations

The following abbreviations are used in this manuscript:

AMKL	Acute megakaryoblastic leukemia
HSCT	Hematopoietic stem cell transplantation
AML	Acute myeloid leukemia
GLILD	Granulomatous lymphocytic interstitial lung disease
MRD	Minimal residual disease
MDS	Myelodysplastic syndrome
CRP	C-reactive protein
CMV	Cytomegalovirus
GvHD	Graft-vs.-host disease
